# The Effect of Alcohol on the Nervous System

**Published:** 1995

**Authors:** Jack H. Mendelson

**Affiliations:** Jack H. Mendelson, M.D., is program director of the Alcohol and Drug Abuse Program and codirector of the Alcohol and Drug Abuse Research Center, McLean Hospital, and professor of psychiatry (neuroscience), Department of Psychiatry, Harvard Medical School, Belmont Massachusetts

**Keywords:** AOD effects (AODE), nervous system, AOD withdrawal syndrome, AOD-related (AODR) seizure, delirium tremens, AOD abstinence

## Abstract

In this special section, *Alcohol Health & Research World* salutes the advances that have been made over the past 25 years in the field of alcohol research. The following pages feature commentaries on important articles published in the field. Each commentary discusses the impact that the article has had on the direction of alcohol research, the changes that have occurred in the field since the article was published, and the future trends that are developing as a result of this research. Much of this research, but not all, has been funded by NIAAA.

These 16 seminal articles were chosen after an arduous selection process. Although the papers represent only a fraction of the important work taking place in the field, they clearly show the direction and scope of alcohol research: They reflect the multidisciplinary nature of alcohol research and the promise that this field holds in reducing the incidence of alcohol-related problems in society.

It is now well accepted that alcohol abuse and dependence are biomedical disorders and are not the result of personal moral turpitude or depravity. However, 40 years ago, the conceptualization of alcohol problems as illness rather than personal deviancy was not well accepted in American society. Scholars have noted that “the transformation of alcoholism from depravity to disease began with a clinical report by two eminent neurologists, Drs. Victor and Adams, in 1953” (Mendelson and Mello 1985, p. 265). These physicians carried out the first systematic evaluations of the alcohol withdrawal syndrome on the nervous system and firmly established that the characteristics of alcohol withdrawal syndrome were an important component of severe alcohol-related illness.

**Figure:**
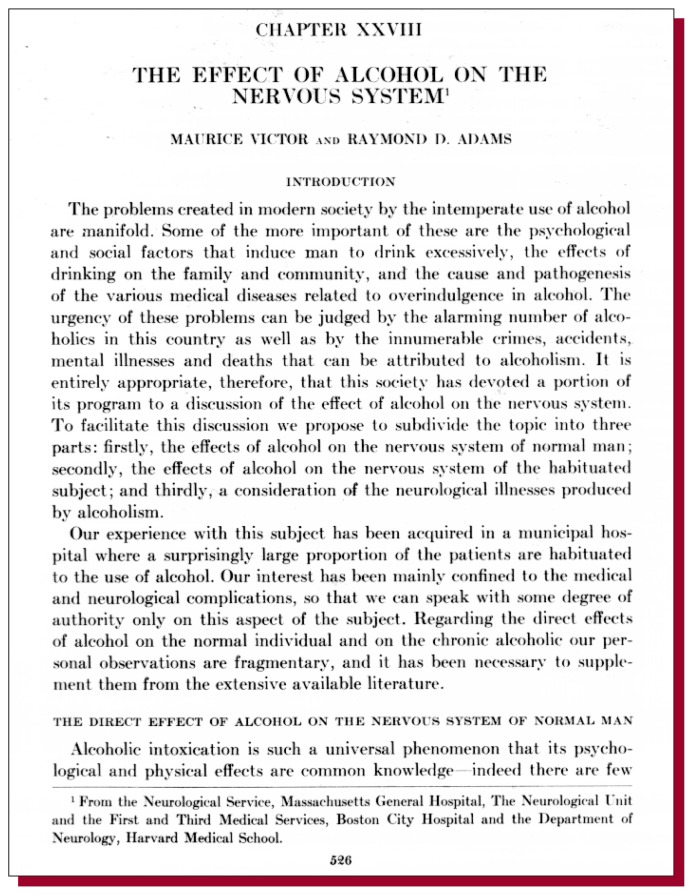
Victor, M., and Adams, R.D. The effect of alcohol on the nervous system. In: *Metabolic and Toxic Diseases of the Nervous System*. Baltimore: Williams and Wilkins Company, 1953. pp. 526–573.

Victor and Adams thoroughly evaluated 226 men admitted to the Boston City Hospital for alcohol-related illness. Beginning at 6 to 8 hours after their last drink, the patients’ behaviors and symptoms were carefully observed and recorded until 72 to 96 hours after cessation of drinking. Victor and Adams observed and described for the first time specific types of alcohol withdrawal symptoms. These symptoms ranged in severity from mild to moderate tremens of the body, which Victor and Adam’s patients described as “the shakes or jitters,” to major seizure disorders and very severe symptoms and behaviors, such as delirium tremens. These basic clinical studies provided the foundation for the development of new treatments, such as the use of medications to reduce the duration and severity of alcohol withdrawal disorders, including the life-threatening consequences of delirium tremens.

Victor and Adams believed that their systematic study of the onset, severity, and duration of the alcohol withdrawal syndrome was consistent with a long tradition of scientific inquiry in the practice of medicine. During scientific society meetings, they often quoted an observation made centuries earlier by Hippocrates, “if the patient be in the prime of life and . . . if from drinking he has trembling hands, it may be well to announce beforehand, either delirium or convulsion” (Mendelson and Mello 1985, p. 266). The careful and imaginative clinical studies carried out by Victor and Adams clearly described a specific relationship between the onset and duration of alcohol abuse and, most importantly, the impact of alcohol abstinence on the type and severity of the alcohol withdrawal syndrome. Victor and Adams stated, “It is difficult to escape the conclusion that the clinical states under discussion depend for their production not only upon the effects of prolonged exposure to alcohol, but temporally, on abstinence from the drug” (Victor and Adams 1953, p. 550).

The pioneering studies performed by Victor and Adams in 1953 also provided the stimulus for other carefully conducted, well-controlled studies. Isbell and colleagues (1955) had previously conducted detailed evaluations of the withdrawal syndromes experienced by persons who were dependent on heroin. Drawing on findings by Victor and Adams, Isbell and his colleagues administered alcohol to heroin-dependent persons and found that after long-term drinking, these individuals developed alcohol withdrawal signs and symptoms. This finding was significant in that it showed that dependence, characterized by withdrawal states, may result after abuse of a wide variety of pharmacologic substances. The influence of Victor and Adams’ work also was evident in a series of multidisciplinary studies carried out by Mendelson and colleagues (see Mendelson 1964) to assess the behavioral and biological aspects of abstinence. These studies included psychiatric and psychological evaluations, tests to assess motor skills and attention, measurements of liver and pancreatic function, and electrolyte studies. Victor was a coinvestigator in these studies in which he used electroencephalographic (EEG) measurements to document changes occurring in the brain during withdrawal.

Prior to the studies by Victor and Adams in 1953, alcohol withdrawal syndrome was considered by many to be the result of problems not specifically related to chronic alcohol intake or cessation of drinking. A variety of causes were invoked to account for severe disorders, including delirium tremens and alcohol seizure disorders. Such causes included poor nutrition, alcohol-induced disorders of metabolism, and even a simpler and more hostile conceptualization–possession of the alcohol consumer by demonic spirits. Victor and Adams, as competent physicians, recorded their observations in a systematic and unbiased manner. Then, based on these observations, they provided the best description of the relationships between the causes and consequences of alcohol dependence. Their characterization of alcohol dependence provided a firm basis for the establishment of alcohol-withdrawal problems as a biomedical as well as biosocial disorder. The studies that they carried out represent not only major contributions for increasing knowledge about alcohol abuse and dependence but serve as an exemplary model for the conduct of clinical investigations in medicine generally. In many ways, the original investigations of Victor and Adams concerning the effect of alcohol on the nervous system highlight the ongoing quest for attainment of excellence in both clinical research and the practice of medicine.
